# Supplementary figures and images for: Comparative analysis of extracellular vesicles from induced and adipose-derived Mesenchymal Stem Cells: Implications for regenerative medicine
Source: PLoS One. 2025 Jun 4;20(6):e0325065. doi: 10.1371/journal.pone.0325065 (PMC12136449; doi:10.1371/journal.pone.0325065)

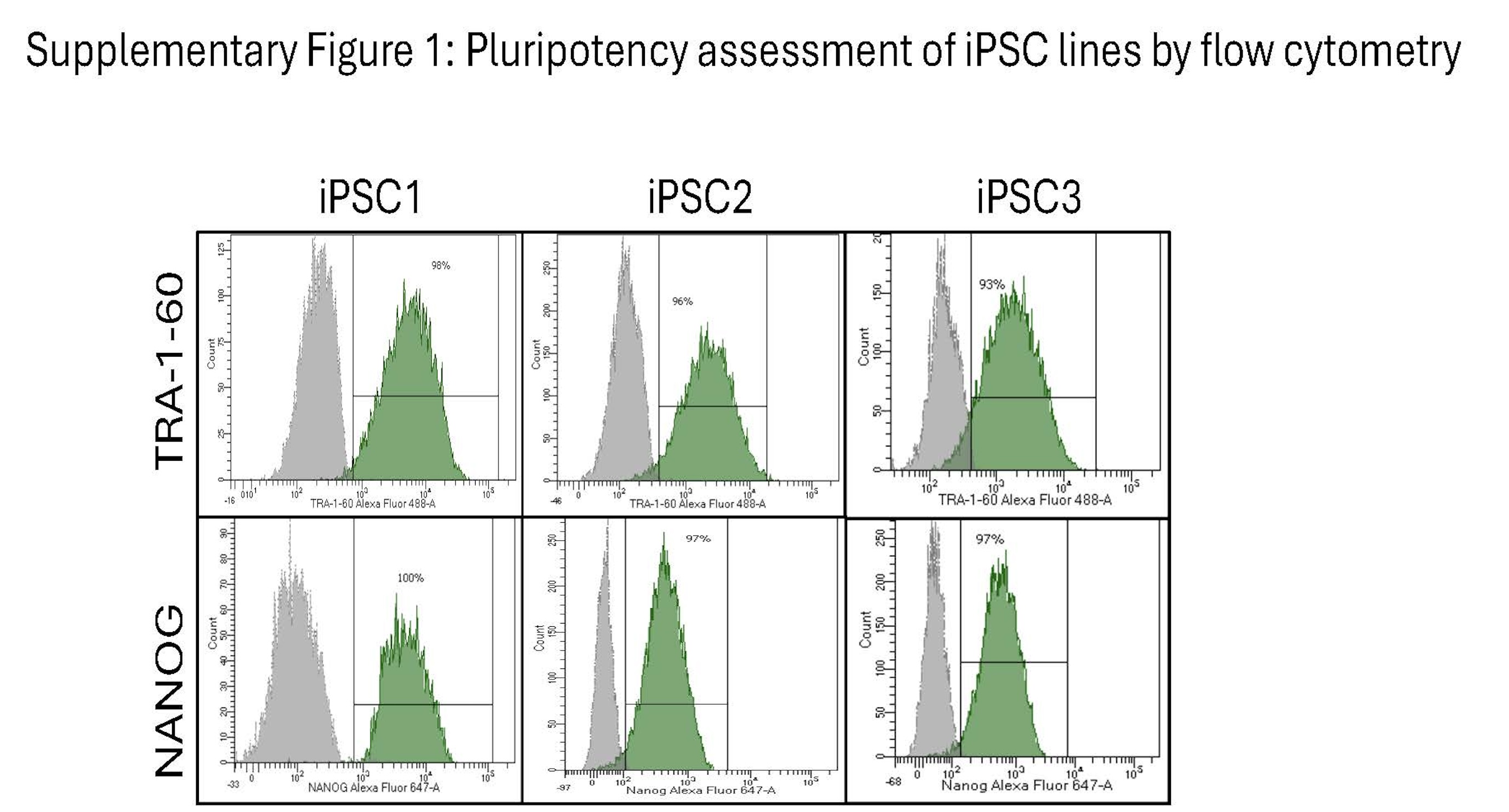

Supplement: S1 Fig — The expression levels of TRA-1–60 and NANOG in three independent iPSC lines (iPSC1, iPSC2, iPSC3) were analyzed using flow cytometry. The green histograms represent the positively stained cells, while the gray histograms indicate the negative control. The percentage of positive cells is shown on each histogram. (JPG) [file pone.0325065.s001.jpg]
