# Supplementary material for: Comparative analysis of extracellular vesicles from induced and adipose-derived Mesenchymal Stem Cells: Implications for regenerative medicine
Source: PLoS One. 2025 Jun 4;20(6):e0325065. doi: 10.1371/journal.pone.0325065 (PMC12136449; doi:10.1371/journal.pone.0325065)
Supplement: Attachment — Submitted filename: feedback for authors.pdf [file pone.0325065.s003.pdf]

Feedback for the Authors:

Ababneh and colleagues have explored the potential of extracellular vesicles (EVs) derived from induced mesenchymal stem cells (iMSCs) as an alternative to EVs from adipose-derived mesenchymal stem cells (ADMSCs) for regenerative medicine. The study compares iMSC-EVs and ADMSC-EVs in terms of size, effects on cell viability, apoptosis, migration, and senescence *in vitro* culture conditions. The authors argue that iMSC-EVs demonstrate a larger particle size and superior performance in enhancing cell viability and migration, while showing comparable effects on senescence and apoptosis reduction to ADMSC-EVs. Despite its innovative premise, the manuscript suffers from methodological inconsistencies, insufficient data to support key claims, unclear controls, and low-quality figure presentation. The manuscript thus requires major revisions to improve clarity, address limitations, and provide additional data where needed. These changes will strengthen the manuscript and enhance its overall impact.

Specific Comments:

1) **EV regenerative properties:** The main goal of the paper was to prove regenerative and wound healing properties of EVs derived from induced MSCs. However, it is not clear if these properties are general for any EVs. It would be thus necessary to include a comparison with control EVs (for example from Human Dermal Fibroblasts used in the experiments) that should lack wound-healing properties to establish the specificity of the observed effects.

2) **Mis-cited references:** The introduction lacks adequate references to support the claims. Mis-cited and irrelevant references make it difficult to verify key statements. Ensure that all references are accurate, recent, and contextually relevant. Here are a couple of examples for consideration:
   a) Introduction, 3rd paragraph. The reference #13 does not support the statement that EVs are superior than MSCs. "*EVs derived from human MSCs (hMSCs) have demonstrated superiority in maintaining similar functions to MSCs and avoiding apparent adverse effects [13].*"
   b) The reference #14 does not contain any mention of EVs. "*Moreover, MSC-derived EVs (MSC-EVs) can replace intact MSCs in tissue repair and regeneration [14].*"
   c) Address inconsistencies in reference style (e.g., "(Van Niel, D'Angelo and Raposo, 2018)" vs. numbered citations).

3) **EV dose used:** The description of EV concentrations (10–50 µg/mL) provided in the methods section is not consistent with the results. Specify which concentration was used for each figure and experiment.

4) **Figure quality and resolution:** All figures need substantial improvement in quality. Microscopy images (Figures 1C, 1D, 5A, 5C) are unclear and need higher resolution. Axes, labels, and legends (e.g., Figure 4B) must be readable and clearly described.

5) **EV size inconsistency:** Discuss the inconsistency and heterogeneity in iMSC-EV size profiles observed in Figure 2C.

6) **Data absent:** Please provide data supporting the following statement for Figure 1. "*Additionally, all iMSC samples were negative for the MSC- negative markers (CD34, CD45, CD14, CD11b, CD79a, CD19, and HLA-DR).*"

7) **Spontaneous apoptosis:** Address the reason for high apoptosis levels in Figure 4C and clarify if the media conditions (e.g., absence of serum) caused this effect. Explore alternative controls like EVs derived from human dermal fibroblasts that do not protect from apoptosis.

8) **Figure 3 description:** Inconsistent description of experiment. Unclear if EVs or cells were stained with Dil.

a) In methods "*iMSCEVs, MSCs-EVs, and PBS were labelled with DiI fluorescent dye selectively stained the plasma membrane and lipids.*" Whereas in Results "*To assess if HDFs can internalize EVs, cells were incubated with Dil as a fluorescent dye and a lipophilic stain, integrated into lipid bilayer membranes, and emitted orange-red fluorescence*".

b) The merged image of ADMSC-EVs does not show the red-Dil labeling staining.

c) The following statement lacks quantitative data for support. Please do quantitative image analysis to provide stronger proof. "*The internalization efficiency of iMSC-EVs was higher than that of ADMSC-EVs.*"
