# Supplementary material for: Comparative analysis of extracellular vesicles from induced and adipose-derived Mesenchymal Stem Cells: Implications for regenerative medicine
Source: PLoS One. 2025 Jun 4;20(6):e0325065. doi: 10.1371/journal.pone.0325065 (PMC12136449; doi:10.1371/journal.pone.0325065)
Supplement: Attachment — Submitted filename: Response Letter.docx [file pone.0325065.s004.docx]

**Response to Reviewer:**

**Comment:** We recognize that you obtained ethical approval from your local ethics committee after you began the study: your ethics approval document is dated 27 May 2021, while participants samples were collected beginning in 01 January 2020 to 01 January 2022. For human subjects research, PLOS ONE requires that authors obtain ethics committee approval before beginning the study, and we do not accept retrospective ethical approval.

**Response:**

We would like to clarify that the collection dates provided were accurate. Our study has received two Institutional Review Board (IRB) approvals: the first in 2019 (IRB-7-2019-7) and the second in 2021 (IRB-CTC/2-2021/06). The 2019 approval was obtained prior to the start of sample collection, and the 2021 approval was issued on May 27, 2021. We apologize for any confusion this may have caused regarding the ethical approval timeline in our previous submission, and we have revised the manuscript to clarify this. And uploaded the two IRB documents on our new submission.

We fully understand the importance of adhering to PLOS ONE's ethical guidelines and assure you that the study received the necessary ethical clearances before participant recruitment began. In response to your request, we have updated the Methods section to include the dates when data were accessed for research purposes, which are now highlighted in green in the revised manuscript.

**Updated Text in Methods:**

“This study adhered to ethical guidelines and was approved by the Institutional Review Board of the University of Jordan and the Cell Therapy Center in 2019 (IRB-7-2019-7) and 2021 (IRB-CTC/2-2021/06). All donors provided signed written informed consent for their participation. Adipose tissue samples were collected from patients undergoing liposuction at the Plastic Surgery Department of Jordan University Hospital (JUH) between January 1, 2020, and January 1, 2022.”

Thank you for considering our manuscript.

**Sincerely,**

Nidaa Ababneh
Corresponding Author
