# Supplementary material for: Comparative analysis of extracellular vesicles from induced and adipose-derived Mesenchymal Stem Cells: Implications for regenerative medicine
Source: PLoS One. 2025 Jun 4;20(6):e0325065. doi: 10.1371/journal.pone.0325065 (PMC12136449; doi:10.1371/journal.pone.0325065)
Supplement: Attachment — Submitted filename: Point-by-point Response 11 Mar 25.docx [file pone.0325065.s005.docx]

PONE-D-24-47284

Comparative Analysis of Extracellular Vesicles from Induced and Adipose-Derived Mesenchymal Stem Cells: Implications for Regenerative Medicine

Please address and incorporate the following queries and suggestions, along with the concerns raised by the reviewer. "The section under this heading “Generation of Embryoid Bodies from iMSCs (EB-iMSCs)” should be divided into two distinct parts to improve clarity and flow. The second part should focus on the protocol for generating embryoid bodies (EBs) from induced Mesenchymal Stem Cells (iMSCs), including a detailed step-by-step process for embryoid body formation. This would include key factors such as cell seeding densities, culture conditions, and the duration of the induction period. It would be helpful to mention the media used for example, MSC differentiation medium used during the process, including any supplements or factors that are crucial for inducing pluripotency or differentiation into multiple germ layers. This would provide readers with a comprehensive understanding of the conditions required to generate functional MSCs. Additionally, to strengthen the claims of regenerative potential of exosome, further assays to assess the regenerative capabilities of exosomes derived from iMSCs should be included.

**Response:** Thank you for your valuable feedback. We have revised the section titled "Generation of Embryoid Bodies from iMSCs (EB-iMSCs)" by dividing it into two distinct parts for better clarity and flow. The first part is the **Ethical Approval and Donor Consent,** and the second part is the **Generation of Induced Mesenchymal Stem Cells (iMSCs) via Embryoid Bodies (EBs)**. The first part provides a clear introduction to the protocol, while the second part includes a detailed, step-by-step process for embryoid body (EB) formation from induced mesenchymal stem cells (iMSCs). We have also incorporated the following details: Cell seeding densities, culture conditions, duration of induction period, media used (e.g., MSC differentiation medium), supplements or factors required for inducing pluripotency or differentiation into multiple germ layers.

We agree that further investigation of the regenerative capabilities of exosomes would largely strengthen the manuscript. However, this would have been tested in a supplemental project that is beyond the funding and logistical potential of the current manuscript. We are keen for the Editor’s understanding that conducting such state-of-the-art work, while essential, is not currently feasible. However, and to accommodate this concern, we expanded the Discussion section by incorporating recent studies demonstrating the regenerative potential of iMSC-derived exosomes in angiogenesis, tissue repair, and immune modulation. Additionally, we have addressed future directions by suggesting additional functional assays that could further validate the therapeutic potential of iMSC-derived exosomes. We hope that these modifications are somewhat satisfactory to this concern.

Regarding the figures, Figures 1C & D, Figure 2D, Figure 3D, and Figures 4A & C appear to be blurred and lack the resolution necessary for clear interpretation. These figures are crucial for supporting the results and should be reprocessed or replaced with higher-quality images to ensure clarity and improve the overall visual presentation. Enhanced clarity in these figures would make the data more convincing and allow for a better understanding of the experimental outcomes."

**Response:** Thank you for your valuable feedback. We appreciate the importance of ensuring that all figures are clear and of high resolution for proper interpretation. We have reprocessed Figures 1C & D, Figure 2 C & D , Figure 3 and Figures 4A & C to enhance their clarity and resolution. We hope the updated figures now meet the journal’s image quality requirements, and the data are presented in a more visually interpretable manner.

Please include the following items when submitting your revised manuscript:

- A rebuttal letter that responds to each point raised by the academic editor and reviewer(s). You should upload this letter as a separate file labeled 'Response to Reviewers'.
- A marked-up copy of your manuscript that highlights changes made to the original version. You should upload this as a separate file labeled 'Revised Manuscript with Track Changes'.
- An unmarked version of your revised paper without tracked changes. You should upload this as a separate file labeled 'Manuscript'.

**Response:** The revised, marked-up and clean, versions of the manuscript have been submitted along with the point-by-point responses to all reviewers’ comments below.

**Response to Editor’s comments**

1. Please ensure that your manuscript meets PLOS ONE's style requirements, including those for file naming. The PLOS ONE style templates can be found at

<https://journals.plos.org/plosone/s/file?id=wjVg/PLOSOne_formatting_sample_main_body.pdf> and

[https://journals.plos.org/plosone/s/file?id=ba62/PLOSOne_formatting_sample_title_authors_affiliations.pdf](https://track.editorialmanager.com/CL0/https:%2F%2Fjournals.plos.org%2Fplosone%2Fs%2Ffile%3Fid=ba62%2FPLOSOne_formatting_sample_title_authors_affiliations.pdf/1/010f01948ba84c68-0f0c1d25-edbb-40ee-a63d-ad447c370592-000000/onORA4Ax591viJFR7mnR75kPm6sLPVJdSuwFeqkAvwY=195)

**Response:** Thank you for your guidance. We have reviewed and updated the manuscript to meet PLOS ONE's style requirements, including file naming.

2. Thank you for stating the following financial disclosure:
“This work was supported by the Deanship of Research at the Jordan University of Science and Technology [grant number 20210398] and the Deanship of Scientific Research at the University of Jordan (141/2020).”

Please state what role the funders took in the study. If the funders had no role, please state: "The funders had no role in study design, data collection and analysis, decision to publish, or preparation of the manuscript."
If this statement is not correct you must amend it as needed.
Please include this amended Role of Funder statement in your cover letter; we will change the online submission form on your behalf.

**Response:** Thank you for your guidance regarding the financial disclosure statement. We confirm that the funders had no role in the study’s design, data collection and analysis, decision to publish, or preparation of the manuscript. Accordingly, we have amended the financial disclosure to read: **"The funders had no role in study design, data collection and analysis, decision to publish, or preparation of the manuscript."**

3. We note that your Data Availability Statement is currently as follows: All relevant data are within the manuscript and its Supporting Information files.

Please confirm at this time whether or not your submission contains all raw data required to replicate the results of your study. Authors must share the “minimal data set” for their submission. PLOS defines the minimal data set to consist of the data required to replicate all study findings reported in the article, as well as related metadata and methods (<https://journals.plos.org/plosone/s/data-availability#loc-minimal-data-set-definition>).

For example, authors should submit the following data:

- The values behind the means, standard deviations and other measures reported;
- The values used to build graphs;
- The points extracted from images for analysis.

Authors do not need to submit their entire data set if only a portion of the data was used in the reported study.

If your submission does not contain these data, please either upload them as Supporting Information files or deposit them to a stable, public repository and provide us with the relevant URLs, DOIs, or accession numbers. For a list of recommended repositories, please see <https://journals.plos.org/plosone/s/recommended-repositories>.

If there are ethical or legal restrictions on sharing a de-identified data set, please explain them in detail (e.g., data contain potentially sensitive information, data are owned by a third-party organization, etc.) and who has imposed them (e.g., an ethics committee). Please also provide contact information for a data access committee, ethics committee, or other institutional body to which data requests may be sent. If data are owned by a third party, please indicate how others may request data access.

**Response:** Thank you for your feedback regarding the Data Availability Statement. We acknowledge the journal’s requirements for sharing the minimal data set. In response to that, we did the following:

1. We revised Data Availability Statement in the manuscript to align with journal policies: “**All relevant data are within the manuscript and its Supporting Information files.**.”

2. Graph Modifications: We have updated all graphical representations to display individual replicate values alongside summary statistics to enhance transparency. To ensure full reproducibility, we have also submitted the GraphPad Prism files containing raw data, statistical analysis details, and figure generation settings.

4. We notice that your supplementary figures are uploaded with the file type 'Figure'. Please amend the file type to 'Supporting Information'. Please ensure that each Supporting Information file has a legend listed in the manuscript after the references list.

**Response:** Thank you for pointing this out. We have resubmitted the supplementary figures under the ‘Supporting Information’ category and ensured that the file has a corresponding legend listed at the end of the manuscript in a section titled “Supporting information” after the references.

**Reviewers' comments:**

Review Comments to the Author

**Reviewer #1:**

Nashwan et al. have performed a comparative analysis of EVs isolated from iMSCs and ADMSCs. The authors need to work on the following comments to improve the manuscript.
1. Authors have demonstrated the role of EVs specifically in terms of mesenchymal cells and the generation of EVs from immune cells has not been discussed. Therefore, the **following references are suggested** to be cited in the third paragraph after reference #12

a. PMID: 36570199

b. PMID: 36713536

c. PMID: 38753658

**Response:** Thank you for your suggestions. We have incorporated the suggested references into the third paragraph of the Introduction section, following reference #12. The suggested references, now numbered as 14, 15, and 16, provide additional context on the generation of EVs from immune cells.

2. Authors are suggested to incorporate these studies, which have indicated the negative role of MSC in bone cancer formation, along with reference 6.

a. PMID: 36769180

**Response:** Thank you for the suggestion. We have incorporated the study (PMID: 36769180) into the manuscript, discussing the negative role of MSCs in bone cancer formation, and it is now numbered as reference #7, alongside with reference #6 as suggested.

3. Authors are suggested to explain the findings of senescence data, if there is a significant difference between the SFM and ADMS-EVs in apoptosis and MTT data then why is it not a case of senescence?

**Response:** Thank you for your important observation. We have updated the Discussion section on senescence to state the following:

“*While we observed non-significant reductions in cellular senescence in HDF cells treated with both iMSC-EVs and ADMSC-EVs, literature suggests that MSC exosomes typically reduce senescence. For instance, Wang et al. showed that human fetal MSC secretome alleviates senescence in adult MSCs by reducing SA-βGal activity and promoting proliferation and osteogenic differentiation [57]. Similarly, MSC-derived supernatants modulate senescence in IL1β-treated osteoarthritis chondrocytes by regulating SA-βGal and reducing γH2AX foci and actin stress fibers [58] . However, our study did not observe significant reductions in the percentage of SA-βGal-positive cells. This discrepancy could be due to differences in experimental conditions, cell types, or the limited range of senescence markers assessed. While both EV types showed a trend toward reducing senescence, these effects were less pronounced than those reported in other studies [59], [60].*”

4. The discussion section is again solely dedicated to mesenchymal cells, it should be broadly discussed in terms of EVs.

**Response:** Thank you for your valuable feedback. In response, we have revised the Discussion section to broaden the scope beyond mesenchymal stem cell-derived EVs (MSC-EVs) and provide a more comprehensive perspective on extracellular vesicles (EVs) from different cell types.

**Reviewer #2:**

Ababneh and colleagues have explored the potential of extracellular vesicles (EVs) derived from induced mesenchymal stem cells (iMSCs) as an alternative to EVs from adipose-derived mesenchymal stem cells (ADMSCs) for regenerative medicine. The study compares iMSC-EVs and ADMSC-EVs in terms of size, effects on cell viability, apoptosis, migration, and senescence in vitro culture conditions. The authors argue that iMSC-EVs demonstrate a larger particle size and superior performance in enhancing cell viability and migration, while showing comparable effects on senescence and apoptosis reduction to ADMSC-EVs. Despite its innovative premise, the manuscript suffers from methodological inconsistencies, insufficient data to support key claims, unclear controls, and low-quality figure presentation. The manuscript thus requires major revisions to improve clarity, address limitations, and provide additional data where needed. These changes will strengthen the manuscript and enhance its overall impact.

**Response**: We highly appreciate the time and effort by the reviewer to deliver this thorough and constructive feedback. We hope that the modifications conducted in the light of these comments have now greatly improved the quality of the work.

Specific Comments:

1) EV regenerative properties: The main goal of the paper was to prove regenerative and wound healing properties of EVs derived from induced MSCs. However, it is not clear if these properties are general for any EVs. It would be thus necessary to include a comparison with control EVs (for example from Human Dermal Fibroblasts used in the experiments) that should lack wound-healing properties to establish the specificity of the observed effects.

**Response:** The reviewer raises a very interesting point. We acknowledge the importance of establishing whether the regenerative and wound-healing properties observed in iMSC-derived EVs are specific or represent a general effect of EVs. While we did not initially include a direct comparison with control EVs, we have now incorporated additional data to the MTT from Human Dermal Fibroblast-derived EVs (HDF-EVs) to provide a broader perspective.
Due to experimental limitations, HDF-EVs were not tested in other functional assays (e.g., apoptosis and wound healing). The MTT assay was chosen as the primary comparison because it allows for a general assessment of EV-induced changes in cell viability, which serves as an indirect measure of regenerative potential. Future studies will be required to further explore the functional effects of HDF-EVs in additional assays, such as migration and apoptosis, to comprehensively assess their role. We are keen on the reviewer’s kind understanding on this issue.

2) Mis-cited references: The introduction lacks adequate references to support the claims. Mis-cited and irrelevant references make it difficult to verify key statements. Ensure that all references are accurate, recent, and contextually relevant. Here are a couple of examples for consideration:
a) Introduction, 3rd paragraph. The reference #13 does not support the statement that EVs are superior than MSCs. “EVs derived from human MSCs (hMSCs) have demonstrated superiority in maintaining similar functions to MSCs and avoiding apparent adverse effects [13].”
b) The reference #14 does not contain any mention of EVs. “Moreover, MSC-derived EVs (MSC-EVs) can replace intact MSCs in tissue repair and regeneration [14].”
c) Address inconsistencies in reference style (e.g., "(Van Niel, D’Angelo and Raposo, 2018)" vs. numbered citations).

**Response:** Thank you for your careful assessment of the citations. We have thoroughly reviewed the Introduction section and made the following revisions to ensure that all references are accurate, recent, and contextually relevant:

- Corrected Mis-Cited References.
- Ensured Citation Consistency.
- Strengthened References in the Introduction, by adding recent references in the introduction and discussion.

**References added to the introduction:**

doi: 10.1093/jleuko/qiae117.

doi: 10.3389/fonc.2022.1042730.

doi: 10.1097/JCMA.0000000000000899.

doi: 10.1007/s13770-023-00557-6.

**References added to the materials and methods:**

doi: 10.1016/j.heliyon.2024.e38857.

**References added to the results:**

doi: 10.1111/febs.16983.

3) **EV dose used**: The description of EV concentrations (10–50 µg/mL) provided in the methods section is not consistent with the results. **Specify which concentration was used** for each figure and experiment.

Response: Thank you for highlighting the inconsistency in EV concentrations. We have carefully reviewed the Methods and Results sections to ensure clarity and consistency regarding the EV dosage used in each experiment.

To clarify, unless otherwise stated, most experiments were conducted using a standardized EV concentration of 50 µg/mL. This is explicitly detailed in various sections of the Methods.

- EV Collection and Purification: “*EVs were isolated, and their concentration was adjusted to 1 mg/mL, ensuring uniform concentration standardization before experimental use. This was achieved using the BCA™ Protein Assay Kit (Thermo Scientific, USA).*”
- EV Staining and Uptake Assay: “*EVs were labeled and used at a defined concentration of approximately 100 µg/mL for uptake experiments, maintaining consistency across different conditions*.”
- In Vitro Assessments (Viability, Apoptosis, Senescence and Wound Healing): “*Cells were treated with 50 µg/mL of iMSC- or ADMSC-derived EVs”,* ensuring that all viability and apoptosis assessments were performed under standardized conditions.

4) Figure quality and resolution: **All figures need substantial improvement in quality**. Microscopy images (Figures 1C, 1D, 5A, 5C) are unclear and need higher resolution. Axes, labels, and legends (e.g., Figure 4B) must be readable and clearly described.

**Response:** Thank you for your comment. To enhance figure clarity, we have enlarged the microscopy images (Figures 1C and 1D) and outlined the wound borders in Figures 5A and 5C to improve visibility and ensure all details are clearly presented. These adjustments improve the readability of cellular structures while maintaining the integrity of the original data.

Additionally, we have reviewed axes, labels, and legends for (Figure 1B, 2B, 4A, 4B, 4E, 5B and 5D) and ensured they are legible and properly formatted for clarity.

We appreciate this feedback and believe these modifications significantly enhance the presentation of our figures.

5) EV size inconsistency: Discuss the **inconsistency and heterogeneity in iMSC-EV** size profiles observed in Figure 2C.

**Response:** Thank you for your insightful comment. EVs are heterogeneous in size, which is a well-documented characteristic. We have now reviewed the introduction and updated the reference in the Introduction (Ref. #12) to support this. Additionally, we acknowledge the limitations of the ultracentrifugation protocol used for exosome isolation. While this method yields a high concentration of exosomes, it can contribute to aggregation, as reported in the literature (Varderidou-Minasian and Lorenowicz, 2020; <https://doi.org/10.7150/thno.40122> ). Although this limitation does not affect the therapeutic relevance of our findings, it highlights the need for alternative or complementary approaches to improve exosome isolation.”

We have now addressed the observed size inconsistency and heterogeneity of iMSC-EVs in Figure 2C within the Discussion section as follows:

“*EVs from both cell types fell within the typical size range of EVs/exosomes (30–200 nm). The heterogeneous sizes observed in both iMSC-EVs and ADMSC-EVs are expected, as they naturally fall within this range. Moreover, iMSC-EVs exhibited extracellular aggregates, which may have resulted from the high concentration of iMSC-EV samples or the ultracentrifugation process, a known limitation of this study. While this method yields a high concentration of small EVs, it can also contribute to aggregation, as reported in the literature [47]. Although this limitation does not affect the therapeutic relevance of our findings, it highlights the need for alternative or complementary approaches to improve EVs isolation. [48].”.*

We hope that these modifications are somewhat satisfactory to your concern.

6) Data absent: Please provide data supporting the following statement for Figure 1. “Additionally, all iMSC samples were negative for the MSC- negative markers (CD34, CD45, CD14, CD11b, CD79a, CD19, and HLA-DR).”

Response: Thank you for your comment. We have now included the supporting data in (Figure 1. a), confirming that all iMSC samples were negative for the MSC-negative markers; *“Additionally, both iMSC and ADMSC samples were negative for the MSC-negative markers (CD34, CD45, CD14, CD11b, CD79a, CD19, and HLA-DR) (Fig 1A and B).*”

7) Spontaneous apoptosis: Address the reason for high apoptosis levels in Figure 4C and clarify if the media conditions (e.g., absence of serum) caused this effect. Explore alternative controls like EVs derived from human dermal fibroblasts that do not protect from apoptosis.

**Response:** Thank you for your insightful comment. To address the concern regarding spontaneous apoptosis, we have included ADMSC cultures as an additional control in (Figure 4. C) and expanded the discussion accordingly. Our findings show that both iMSC-EVs and ADMSC-EVs significantly reduced apoptosis in HDFs and ADMSCs compared to the SFM control, indicating a protective effect. This suggests that the observed apoptosis in the control group is likely due to serum deprivation rather than an intrinsic EV effect.

To clarify this, we have updated the Discussion to explicitly highlight the impact of serum deprivation and its connection to MTT viability assay results. While HDF-derived EVs were not tested in apoptosis assays, we acknowledge their relevance as a non-protective control and suggest including them in future studies as follows:

*“When linked with the MTT assay results, this finding may indicate that the observed outcomes could be attributed to serum deprivation rather than the EV treatments. However, further studies are needed to investigate in more depth the mechanism behind these effects. Despite this limitation, our findings are in line with previous studies showing that MSC-derived EVs can inhibit apoptosis in neuronal cells, likely due to their cargo of anti-apoptotic proteins. Similarly, hUCMSC-EVs have been reported to provide hepatoprotection against acetaminophen (APAP)-induced toxicity, partly by inhibiting oxidative stress-mediated apoptosis via ERK1/2 and PI3K/Akt signaling [55], [56]. These studies highlight the potential of stem cell-derived EVs as modulators of apoptosis and cell survival, although further mechanistic investigations are necessary to confirm their direct effects in our experimental model.”.*

8) Figure 3 description: Inconsistent description of the experiment. It is unclear whether EVs or cells were stained with Dil.

**Response:** Thank you for your comment. We have revised Figure 3 legend and updated the Methods section to explicitly clarify that DiI was used to stain EV membranes, not the entire cells. These changes ensure consistency across the manuscript and Figure descriptions; *“DiI, a lipophilic dye, was used to stain EV membranes before incubation with HDFs”.*

a) In methods “iMSCEVs, MSCs-EVs, and PBS were labelled with DiI fluorescent dye selectively stained the plasma membrane and lipids.” Whereas in Results “To assess if HDFs can internalize EVs, **EVs were incubated with Dil** as a fluorescent dye and a lipophilic stain, integrated into lipid bilayer membranes, and emitted orange-red fluorescence”.

Response**:** Thank you for your comment. We have revised the Methods and Results sections to ensure consistency in describing DiI labeling and its role as a lipophilic dye that integrates into EV membranes. We hope that these modifications address your concern:

*“iMSC-EVs or ADMSC-EVs were labeled with 5 μL of 10 μM DiI, a lipophilic dye that selectively stains EV membranes by integrating into their lipid bilayer.”*

b) The merged image of ADMSC-EVs does not show the red-Dil labeling staining.

**Response:** Thank you for your observation. We have updated the figure to ensure that the red-DiI labeling of ADMSC-EVs is clearly visible in the merged image. This was achieved by adjusting the imaging settings to enhance fluorescence visibility without altering the original data.

c) The following statement lacks quantitative data for support. Please do quantitative image analysis to provide stronger proof. “The internalization efficiency of iMSC-EVs was higher than that of ADMSC-EVs.”

**Response:** Thank you for your comment. After re-evaluating the images, we realized that the originally selected ADMSC-EVs figures were not fully representative, as the uptake appeared visually less apparent. We have now replaced them with more representative images to better reflect our findings.
